# Antioxidant, Hypoglycemic and Molecular Docking Studies of Methanolic Extract, Fractions and Isolated Compounds from Aerial Parts of *Cymbopogon citratus* (DC.) Stapf

**DOI:** 10.3390/molecules27092858

**Published:** 2022-04-30

**Authors:** Hanlei Wang, Ran Zhang, Kun Zhang, Xuelin Chen, Yumei Zhang

**Affiliations:** 1 Key Laboratory of Tropical Plant Resource and Sustainable Use, Xishuangbanna Tropical Botanical Garden, Chinese Academy of Sciences, No.88 Xuefu Road, Kunming 650223, China; wanghanlei@xtbg.ac.cn (H.W.); zhangran213@yeah.net (R.Z.); zhangkun@xtbg.ac.cn (K.Z.); chenxuelin@xtbg.ac.cn (X.C.); 2 University of Chinese Academy of Sciences, Beijing 100049, China

**Keywords:** *Cymbopogon citratus*, chemical composition, antioxidant, *α*-glucosidase, glucose uptake

## Abstract

Traditionally, *Cymbopogon citratus* is used to treat a variety of ailments, including cough, indigestion, fever, and diabetes. The previous chemical and bioactive research on *C. citratus* mainly focused on its volatile oil. In this study, 20 non-volatile known compounds were isolated from the dried aerial part of *C. citratus*, and their structures were elucidated by MS, NMR spectroscopy, and comparison with the published spectroscopic data. Among them, 16 compounds were reported for the first time from this plant. The screening results for antioxidant and *α*-glucosidase inhibitory activities indicated that compounds caffeic acid (**5**), 1-*O*-p-coumaroyl-3-*O*-caffeoylglycerol (**8**), 1,3-*O*-dicaffeoylglycerol (**9**) and luteolin-7-*O*-*β*-D-glucopyranoside (**12**) had potent antioxidant capacities, with IC_50_ values from 7.28 to 14.81 μM, 1.70 to 2.15 mol Trolox/mol and 1.31 to 2.42 mol Trolox/mol for DPPH, ABTS, and FRAP, respectively. Meanwhile, compounds **8** and **9** also exhibited significant inhibitory activities against *α*-glucosidase, with IC_50_ values of 11.45 ± 1.82 μM and 5.46 ± 0.25 μM, respectively, which were reported for the first time for their *α*-glucosidase inhibitory activities. The molecular docking result provided a molecular comprehension of the interaction between compounds (**8** and **9**) and *α*-glucosidase. The significant antioxidant and *α*-glucosidase inhibitory activities of compounds **8** and **9** suggested that they could be developed into antidiabetic drugs because of their potential regulatory roles on oxidative stress and digestive enzyme.

## 1. Introduction

Diabetes mellitus is one of the most serious metabolic diseases in the world, with a high incidence [1]. It is characterized either by insufficient insulin production or insulin resistance caused by genetic and environmental factors, such as obesity, oxidative stress, incorrect dietary components [2,3]. At present, there is a variety of antidiabetic drugs in clinical use, but most of the synthetic antidiabetic drugs have limited therapeutic efficacy and are accompanied by many side effects, such as hypoglycemia, gastrointestinal side effects, and weight gain [4,5]. Meanwhile, natural products have a long history of being used to treat diabetes. Therefore, it might be of great research value to find high-efficiency and low-toxicity hypoglycemic components from traditional antidiabetic plants.

*Cymbopogon citratus* (*C. citratus*), which is also known as lemongrass, has a fragrant taste and belongs to the Gramineae family. It is a perennial herbaceous plant that grows mainly in tropical and subtropical regions. It has a very wide range of uses, such as seasoning, medicine, and tea [6]. As a folk medicine, *C. citratus* can be used to treat inflammation, cough, indigestion, flu, fever, diabetes, and malaria [7,8]. Furthermore, the decoction of *C. citratus* is commonly used to treat diabetes in Thailand [7]. Previous biological studies of *C. citratus* have reported that its extracts exhibit diverse therapeutic properties, including hypoglycemic [9], antioxidant [10], antimicrobial [11] and anti-inflammatory activities [12]. The research by Bharti et al. confirmed that *C. citratus* essential oil could improve the abnormal metabolism of blood glucose, insulin and lipid in Poloxamer 407-induced Wistar rats with type 2 diabetes via increasing the number of *β* cells and promoting the secretion of GLP-1 [9]. In addition, *C. citratus* extracts have been proved to possess certain hypoglycemic effects by inhibiting α-glucosidase and *α*-amylase activities [13].

In recent years, most research on *C. citratus* has focused on its essential oil. There is about 1–2% essential oil in its leaves [14]. The main components of *C. citratus* essential oil are limonene, citral, myrcene, geranial, neral and citronellal [15,16], and citral is the crucial ingredient of *C. citratus* flavor [17]. However, the other chemical constituents of *C. citratus* and their bioactivities are rarely researched, which might be important for its hypoglycemic function.

*α*-Glucosidase is an important target in researching hypoglycemic drugs. It can catalyze the hydrolysis of *α*-1.4-glucosidase, which leads to the hydrolysis of oligosaccharides such as maltose and sucrose in the small intestine [18]. Thus, the inhibition of *α*-glucosidase activity can slow down glucose production and absorption, and then reduce postprandial blood glucose. The molecular docking study can be used to predict the binding sites and modes of small-molecule compounds to α-glucosidase [19]. Oxidative stress occurs when the production of free radicals exceeds the cell’s inherent antioxidant defense system, which plays an important role in the occurrence and development of diabetes and its complications [20]. Antioxidants play a strong role in resisting oxidative stress [21]. Therefore, natural antioxidants could be effective in preventing diabetes. The 3T3-L1 cell line is generally deemed to be a mature and classical cell line used to study glucose metabolism, adipocyte differentiation, and insulin signal transduction in vitro [22]. Therefore, we studied the hypoglycemic activity of *C. citratus* via assaying antioxidant capacity, glycosidase inhibitory activity, and glucose uptake rate.

In this study, chemical constituents of the methanol extract of *C. citratus* were isolated and identified. Furthermore, the antioxidant capacity, *α*-glucosidase inhibitory activity and the glucose uptake rate of crude extracts and compounds were evaluated. Our work reported the inhibitory activity of 1-*O*-p-coumaroyl-3-*O*-caffeoylglycerol (**8**) and 1,3-*O*-dicaffeoylglycerol (**9**) against *α*-glucosidase for the first time. Meanwhile, the potent antioxidant capacities of compounds **8** and **9** suggested that they are potential natural candidate drugs to inhibit the *α*-glucosidase activity and oxidative stress in diabetes.

## 2. Results and Discussion

### 2.1. Structure Elucidation

**Figure 1:**
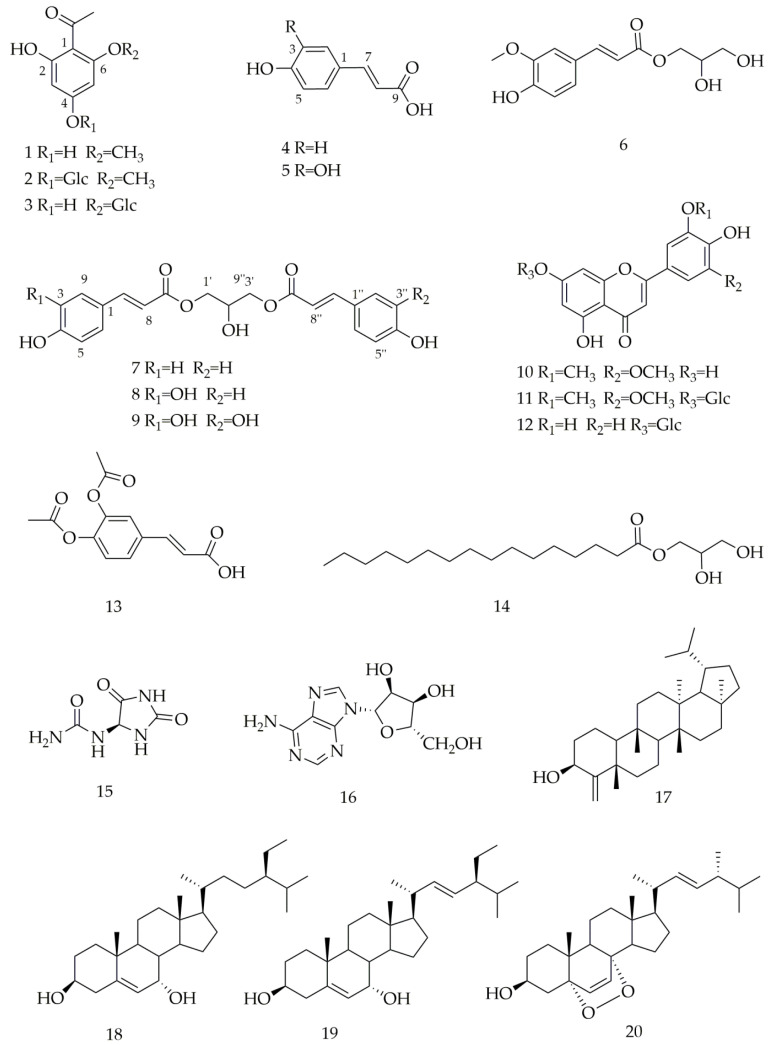
Chemical structures of isolated compounds from *C. citratus*.

Twenty known compounds (Figure 1) were isolated and identified from the dried aerial part of *C. citratus*, including 4,6-dihydroxy-2-methoxyacetophenone (**1**) [23], 4-*O*-*β*-D-glucopyranosyl-2-hydroxy-6-methoxyacetophenone (**2**) [24], 2,4,6-trihydroxy- acetophenone 2-*O*-*β*-D-glucoside (**3**) [25], p-coumaric acid (**4**) [26], caffeic acid (**5**) [27], 1-*O*-feruloylglycerol (**6**) [28], 1,3-*O*-di-p-coumaroylglycerol (**7**) [28], 1-*O*-p-coumaroyl- 3-*O*-caffeoylglycerol (**8**) [29], 1,3-*O*-dicaffeoylglycerol (**9**) [29], tricin (**10**) [30], trcin-7-*O*-*β*-D-glucopyranose (**11**) [31], luteolin-7-*O*-*β*-D-glucopyranoside (**12**) [32], 3,4-diacetoxycinnamic acid (**13**) [33], glyceroyl monopalmitate (**14**) [34], allantion (**15**) [35], adenosine (**16**) [36], cymbopogonol (**17**) [37], 7*α*-hydroxysitosterol (**18**) [38], stigmasta-5,22-diene-3*β*,7*α*-diol (**19**) [39], ergosterol endoperoxide (**20**) [38]. Among them, p-coumaric acid (**4**) was identified by Filipa Tavares et al. from the dried leaves of *C. citratus*, using HPLC-PDA/ESI-MS^n^ [40]. Caffeic acid (**5**) was isolated from dried leaves of *C. citratus* by XiaoLi Bao et al. [41]. Luteolin-7-*O*-*β*-D-glucopyranoside (**12**) was isolated from the dry leaves of *C. citratus* by Pedro H.O. Borges. [8]. Cymbopogonol (**17**) was first isolated from the leaf wax of *C. citratus* by Steven W. Hanson [42]. Except for these four compounds, other compounds were isolated from this species for the first time.

**Compound 1.** C_9_H_10_O_4_, ESI-MS: *m*/*z* 181 [M-H]^–^. ^1^H-NMR (600 MHz, CD_3_COCD_3_) *δ*_H_: 6.03 (1H, d, *J* = 2.2 Hz, H-3), 5.95 (1H, d, *J* = 2.2 Hz, H-5), 3.90 (3H, s, OCH_3_), 2.55 (3H, s, COCH_3_). ^13^C-NMR (150 MHz, CD_3_COCD_3_) *δ*_C_: 106.3 (C-1), 168.7 (C-2), 97.0 (C-3), 166.2 (C-4), 92.1 (C-5), 165.1 (C-6), 203.9 (C=O), 33.4 (COCH_3_), 56.5 (OCH_3_).

**Compound 2.** C_15_H_20_O_9_, ESI-MS: *m*/*z* 367 [M + Na]^+^. ^1^H-NMR (600 MHz, pyridine-d_5_) *δ*_H_: 6.73 (1H, d, *J* = 2.3 Hz, H-3), 6.39 (1H, d, *J* = 2.3 Hz, H-5), 5.77 (1H, d, J = 7.4 Hz, H-1′), 3.71 (3H, s, OCH_3_), 2.54 (3H, s, COCH_3_). ^13^C-NMR (150 MHz, pyridine-d_5_) *δ*_C_: 107.5 (C-1), 167.8 (C-2), 97.9 (C-3), 165.4 (C-4), 92.7 (C-5), 163.9 (C-6), 204.0 (C=O), 33.5 (COCH_3_), 56.2 (OCH3), 101.5 (C-1′), 74.8 (C-2′), 78.6 (C-3′), 71.3 (C-4′), 79.3 (C-5′), 62.4 (C-6′).

**Compound 3.** C_14_H_18_O_9_, ESI-MS: *m*/*z* 353 [M + Na]^+^. ^1^H-NMR (800 MHz, pyridine-d _5_) *δ*_H_: 6.88 (s, 1H, H-3), 6.58 (1H, d, *J* = 6.4 Hz, H-5), 5.68 (d, *J* = 6.4 Hz, 1H, H-1′), 2.96 (s, 3H, CH_3_). ^13^C-NMR (200 MHz, pyridine-d_5_) *δ*_C_: 106.8 (C-1), 163.1 (C-2), 98.7 (C-3), 168.4 (C-4), 96.0 (C-5), 167.5 (C-6), 204.3 (C=O), 34.1 (COCH3), 102.8 (C-1′), 75.2 (C-2′), 79.5 (C-3′), 71.5 (C-4′), 79.6 (C-5′), 62.7 (C-6′).

**Compound 4.** C_9_H_8_O_3_, ESI-MS: *m*/*z* 163 [M-H]^–^. ^1^H-NMR (600 MHz, CD_3_OD) *δ*_H_: 7.58 (1H, d, *J* = 15.9 Hz, H-7), 7.44 (2H, d, *J* = 8.6 Hz, H-2, 6), 6.80 (2H, d, *J* = 8.6 Hz, H-3, 5), 6.30 (1H, d, *J* = 15.9 Hz, H-8). ^13^C-NMR (150 MHz, CD_3_OD) *δ*_C_: 127.5 (C-1), 131.2 (C-2), 116.9 (C-3), 161.3 (C-4), 116.9 (C-5), 131.2 (C-6), 146.5 (C-7), 115.9 (C-8), 171.5 (C-9).

**Compound 5.** C_9_H_8_O_4_, ESI-MS: *m*/*z* 181 [M + H]^+^, ^1^ H-NMR (500 MHz, CD_3_OD) *δ*_H_: 7.52 (1H, d, *J* = 15.9 Hz, H-7), 7.03 (1H, d, *J* = 2.1 Hz, H-2), 6.92 (1H, dd, *J* = 8.2, 2.1 Hz, H-6), 6.77 (1H, d, *J* = 8.2 Hz, H-5), 6.21 (1H, d, *J* = 15.9 Hz, H-8). ^13^C-NMR (125 MHz, CD_3_OD) *δ*_C_: 127.8 (C-1), 115.1 (C-2), 146.8 (C-3), 149.4 (C-4), 116.5 (C-5), 122.8 (C-6), 147.0 (C-7), 115.5 (C-8), 171.1 (C-9).

**Compound 6.** C_13_H_16_O_6_, ESI-MS: *m*/*z* 291 [M + Na]^+^. ^1^H-NMR (600 MHz, CD_3_OD) *δ*_H_: 7.73 (1H, d, *J* = 15.9 Hz, H-7), 7.27 (1H, d, *J* = 1.9 Hz, H-2), 7.16 (1H, dd, *J* = 8.2, 1.9 Hz, H-6), 6.89 (1H, d, *J* = 8.2 Hz, H-5), 6.28 (1H, d, *J* = 15.9 Hz, H-8), 4.34 (1H, dd, *J* = 11.4, 4.3 Hz, H-1′a), 4.25 (1H, d, *J* = 11.4, 6.3 Hz, H-1′b), 3.96 (3H, s, -OCH3), 3.82 (1H, t, *J* = 5.4 Hz H-2′), 3.68 (2H, t, *J* = 5.4 Hz, H-3′). ^13^C-NMR (150 MHz, CD_3_OD) *δ*_C_: 127.8 (C-1), 111.8 (C-2), 150.8 (C-3), 149.5 (C-4), 116.6 (C-5), 124.3 (C-6), 147.2 (C-7), 115.4 (C-8), 169.3 (C-9), 66.7 (C-1′), 71.4 (C-2′), 64.2 (C-3′), 56.6 (OCH3).

**Compound 7.** C_21_H_20_O_7_. ESI-MS: *m*/*z* 407 [M + Na]^+^. ^1^H-NMR (600 MHz, CD_3_COCD_3_) *δ*_H_: 7.66 (2H, d, *J* = 16.1 Hz, H-7, 7″), 7.56 (4H, d, *J* = 8.6 Hz, H-2, 2″, 6, 6″), 6.90 (4H, d, *J* = 8.6 Hz, H-3, 3″, 5, 5″), 6.39 (2H, d, *J* = 16.1 Hz, H-8, 8″), 4.57 (1H, d, *J* = 5.4 Hz, H-2′), 4.28 (4H, m, H-1′, 3′). ^13^C-NMR (150 MHz, CD_3_COCD_3_) *δ*_C_: 127.3 (C-1, 1″), 131.4 (C-2, 2″), 117.1 (C-3, 3″), 161.1 (C-4, 4″), 117.1 (C-5, 5″), 131.4 (C-6, 6″), 146.1 (C-7, 7″), 115.6 (C-8, 8″), 167.8 (C-9, 9″), 66.4 (C-1′), 68.6 (C-2′), 66.4 (C-3′).

**Compound 8.** C_21_H_20_O_8_, ESI-MS: *m*/*z* 423 [M + Na]^+^. ^1^H-NMR (600 MHz, CD_3_COCD_3_) *δ*_H_: 7.66 (1H, d, *J* = 16.0 Hz, H-7″), 7.59 (1H, d, *J* = 16.0 Hz, H-7), 7.56 (2H, d, *J* = 8.6 Hz, H-2″, 6″), 7.18 (1H, d, J = 1.9 Hz, H-2), 7.06 (1H, dd, *J* = 8.2, 1.9 Hz, H-6), 6.89 (2H, dd, *J* = 8.6 Hz, H-3″, 5″), 6.87 (1H, d, *J* = 8.2 Hz, H-5), 6.39 (1H, d, *J* = 16.0 Hz, H-8″), 6.32 (1H, d, *J* = 16.0 Hz, H-8), 4.27 (4H, m, H-1′, 3′), 4.19 (1H, m, H-2′). ^13^C-NMR (150 MHz, CD_3_COCD_3_) *δ*_C_: 127.4 (C-1), 131.4 (C-2), 116.8 (C-3), 161.0 (C-4), 116.8 (C-5), 131.4 (C-6), 146.7 (C-7), 115.7 (C-8), 167.8 (C-9), 66.4 (C-1′), 68.7 (C-2′), 66.4 (C-3′), 128.0 (C-1″), 115.6 (C-2″), 149.2 (C-3″), 146.5 (C-4″), 116.8 (C-5″), 123.0 (C-6″), 146.1 (C-7″), 115.7 (C-8″), 167.8 (C-9″).

**Compound 9.** C_21_H_20_O_9_, ESI-MS: *m*/*z* 439 [M + Na]^+^. ^1^H-NMR (800 MHz, pyridine-d_5_) *δ*_H_: 7.97 (2H, d, *J*=15.8 Hz, H-7, 7″), 7.57 (2H, d, *J* = 1.7 Hz, H-2, 2″), 7.21 (2H, d, *J* = 8.1 Hz, H-5, 5″), 7.12 (2H, dd, *J* = 8.1, 1.7 Hz, H-6, 6″), 6.58 (2H, d, *J* = 15.8 Hz, H-8, 8″). ^13^C-NMR (200 MHz, pyridine-d_5_) *δ_C_*: 127.2 (C-1, 1″), 116.2 (C-2, 2″), 150.5 (C-3, 3″), 146.5 (C-4, 4″), 117.0 (C-5, 5″), 122.5 (C-6, 6″), 146.5 (C-7, 7″), 115.0 (C-8, 8″), 167.8 (C-9, 9″), 66.6 (C-1′), 68.3 (C-2′), 66.6 (C-3′).

**Compound 10.** C_17_H_14_O_7_, ESI-MS: *m*/*z* 329 [M-H] ^–^. ^1^H-NMR (500 MHz, pyridine-d_5_) *δ*_H_: 7.44 (2H, s, H-2′, 6′), 7.03 (1H, s, H-3), 6.89 (1H, d, *J* = 2.1 Hz, H-6), 6.76 (1H, d, *J* = 2.1 Hz, H-8), 3.86 (6H, s, OCH_3_). ^13^C-NMR (125 MHz, pyridine-d_5_) *δ*_C_: 164.7 (C-2), 104.6 (C-3), 182.8 (C-4), 163.2 (C-5), 100.1 (C-6), 166.0 (C-7), 95.1 (C-8), 158.7 (C-9), 105.0 (C-10), 121.4 (C-1′), 105.3 (C-2′, 6′), 149.4 (C-3′, 5′), 164.7 (C-4′), 56.6 (OCH_3_).

**Compound 11.** C_23_H_24_O_12_, ESI-MS: *m*/*z* 515 [M + Na]^+^. ^1^H-NMR (600 MHz, pyridine-d_5_) *δ*_H_: 7.45 (2H, s, H-2′, 6′) 7.20 (1H, d, *J* = 2.1 Hz, H-8), 7.06 (1H, s, H-3), 6.90 (1H, d, *J* = 2.1 Hz, H-6), 5.82 (1H, d, J = 6.9 Hz, H-1″), 3.89 (6H, s, OCH_3_). ^13^C-NMR (150 MHz, pyridine-d_5_) *δ*_C_: 165.4 (C-2), 104.9 (C-3), 183.2 (C-4), 162.9 (C-5), 101.2 (C-6), 164.5 (C-7), 95.9 (C-8), 158.3 (C-9), 107.0 (C-10), 121.2 (C-1′), 105.5 (C-2′, 6′), 150.5 (C-3′, 5′), 142.6 (C-4′), 56.9 (OCH3), 101.1 (C-1″), 75.2 (C-2″), 79.6 (C-3″), 71.4 (C-4″), 79.0 (C-5″), 62.6 (C-6″).

**Compound 12.** C_21_H_20_O_11_, ESI-MS: *m*/*z* 447 [M-H]^–^. ^1^H-NMR (600 MHz_,_ pyridine-d*5*) *δ*_H_: 7.92 (1H, d, *J* = 2.0, H-2′), 7.53 (1H, dd, *J* = 8.3, 2.0 Hz, H-6′), 7.29 (1H, d, *J* = 8.3 Hz, H-5′), 7.02 (1H, d, *J* = 1.9 Hz, H-8), 6.96 (1H, s, H-3), 6.87 (1H, d, *J* = 1.9 Hz, H-6), 5.86 (1H, d, *J* = 7.7 Hz, H-1″). ^13^C NMR (150 MHz, C_5_D_5_N_5_) *δ*_C_: 165.7 (C-2), 104.6 (C-3), 183.3 (C-4), 163.0 (C-5), 101.0 (C-6),164.4 (C-7), 95.7 (C-8),158.3 (C-9), 107.0 (C-10), 123.1 (C-1′), 115.1 (C-2′), 148.3 (C-3′), 152.4 (C-4′), 117.3 (C-5′), 120.1 (C-6′), 102.2 (C-1″), 75.3 (C-2″), 78.9 (C-3″), 71.5 (C-4″), 79.7 (C-5″), 62.8 (C-6″).

**Compound 13.** C_13_H_12_O_6_. ESI-MS: *m*/*z* 287 [M + Na]^+^. ^1^H-NMR (500 MHz, CD_3_OD) *δ*_H_: 7.63 (1H, d, *J* = 16.0 Hz, H-7), 7.51 (1H, dd, *J* = 8.3, 2.0 Hz, H-6), 7.49 (1H, d, *J* = 2.0 Hz, H-2), 7.25 (1H, d, *J* = 8.3 Hz, H-5), 6.47 (1H, d, *J* = 16.0 Hz, H-8), 2.27 (6H, d, *J* = 4.6 Hz, COCH_3_). ^13^C-NMR (125 MHz, CD_3_OD) *δ*_C_: 169.0 (C-9), 120.6 (C-8), 144.3 (C-7), 134.7 (C-1), 125.1 (C-2), 144.1 (C-3), 145.1 (C-4), 125.1 (C-5), 127.5 (C-6), 167.0 (COCH_3_), 169.9 (COCH_3_), 20.4 (COCH_3_).

**Compound 14.** C_19_H_38_O_4_, ESI-MS: *m*/*z* 353 [M + Na]^+^. ^1^H-NMR (500 MHz, CDCl_3_) *δ*_H_: 4.20 (1H, dd, *J* = 11.7, 4.6 Hz, H-1a), 4.15 (1H, dd, *J* = 11.7, 6.2 Hz, H-1b), 3.93 (1H, m, H-2), 3.70 (1H, dd, *J* = 11.4, 3.4 Hz, H-3a), 3.60 (1H, dd, *J* = 11.4, 5.8 Hz, H-3b), 2.35 (2H, t, *J* = 7.6 Hz, H-2′), 0.88 (3H, t, *J* = 7.0 Hz, H-16′). ^13^C-NMR (125 MHz, CDCl_3_) *δ*_C_: 174.6 (C-1), 34.4 (C-2), 32.1 (C-3), 29.9-22.7 (C-4~15), 14.4 (C-16), 65.4(C-1′), 70.5 (C-2′), 63.5 (C-3′).

**Compound 15.** C_4_H_6_N_4_O_3_, ESI-MS: *m*/*z* 157 [M-H]^–^. ^1^H-NMR (600 MHz, DMSO-d_6_) *δ*_H_: 10.55 (1H, s, NH-3), 8.07 (1H, s, NH-1), 6.89 (1H, d, *J* = 8.2 Hz, NH-4), 5.80 (2H, s, NH_2_-6), 5.24 (1H, d, *J* = 8.2 Hz, H-4). ^13^C-NMR (150 MHz, DMSO-d_6_) *δ*_C_: 156.8 (C-2), 62.4 (C-4), 173.37 (C-5), 157.4 (C-6).

**Compound 16.** C_10_H_13_N_5_O_4_, ESI-MS: *m*/*z* 268 [M + H]^+^. ^1^H-NMR (600 MHz, pyridine-d_5_) *δ*_H_: 8.67 (1H, s, H-8), 8.44 (2H, s, -NH_2_), 6.75 (1H, d, *J* = 5.9 Hz, H-1′), 4.33 (1H, d, *J* = 12.0 Hz, H-5′a), 4.15 (1H, d, *J* = 12.0 Hz, H-5′b). ^13^C-NMR (150 MHz, pyridine-d_5_) *δ*_C_: 153.8 (C-2), 150.7 (C-4), 122.0 (C-5), 158.2 (C-6), 141.0 (C-8), 91.3 (C-1′), 76.0 (C-2′), 72.9 (C-3′), 88.3 (C-4′), 63.5 (C-5′).

**Compound 17.** C_30_H_50_O_,_ ESI-MS: *m*/*z* 449 [M + Na]^+^. 1H-NMR (500 MHz, CDCl_3_) *δ*_H_: 4.84 (1H, d, *J* = 1.4 Hz, H-23a), 4.78 (1H, d, *J* = 1.4 Hz, H-23b), 4.31 (1H, t, *J* = 2.9 Hz, H-3), 1.23 (3H, s, H-24), 0.94 (3H, s, H-25), 0.87 (3H, s, H-26), 0.84 (3H, s, H-27), 0.96 (3H, s, H-28), 0.88 (3H, d, *J* = 6.6 Hz, H-29), 0.89 (3H, d, *J* = 6.6 Hz, H-30). 13C-NMR (125 MHz, CDCl_3_) *δ*_C_: 15.7 (C-1), 34.9 (C-2), 74.8 (C-3), 161.2 (C-4), 40.1 (C-5), 40.3 (C-6), 15.7 (C-7), 50.0 (C-8), 37.9 (C-9), 59.4 (C-10), 34.9 (C-11), 30.2 (C-12), 39.0 (C-13), 39.7 (C-14), 29.3 (C-15), 32.4 (C-16), 39.73 (C-17), 54.2 (C-18), 48.0 (C-19), 27.7 (C-20), 43.0 (C-21), 30.2 (C-22), 109.3 (C-23), 23.8 (C-24), 18.5 (C-25), 16.5 (C-26), 15.8 (C-27), 33.54 (C-28), 21.0 (C-29), 23.4 (C-30).

**Compound 18.** C_29_H_50_O_2_, ESI-MS: *m*/*z* 453 [M + Na]^+^. ^1^H-NMR (500 MHz, CDCl_3_) *δ*_H_: 5.60 (1H, dd, *J* = 5.3, 1.7 Hz, H-6), 3.85 (1H, s, H-7), 3.59 (1H, m, H-3), 1.00 (3H, s, H-19), 0.93 (3H, d, *J* = 6.5 Hz, H-21), 0.84 (3H, d, *J* = 6.5 Hz, H-27), 0.82 (3H, d, *J* = 6.8 Hz, H-26), 0.68 (3H, s, H-18). ^13^C-NMR (125 MHz, CDCl_3_) *δ*_C_: 37.0 (C-1), 31.4 (C-2), 71.4 (C-3), 42.0 (C-4), 146.3 (C-5), 123.9 (C-6), 65.4 (C-7), 39.2 (C-8), 42.3 (C-9), 37.4 (C-10), 20.7 (C-11), 39.2 (C-12), 42.2 (C-13), 49.4 (C-14), 24.3 (C-15), 28.3 (C-16), 55.7 (C-17), 11.7 (C-18), 18.3 (C-19), 36.1 (C-20), 18.8 (C-21), 33.9 (C-22), 25.9 (C-23), 45.8 (C-24), 29.1 (C-25), 19.0 (C-26), 19.8 (C-27), 23.1 (C-28), 12.0 (C-29).

**Compound 19.** C_29_H_48_O_2_. ESI-MS: *m*/*z* 451 [M + Na]^+^. ^1^H-NMR (800 MHz, CDCl_3_) *δ*_H_: 5.60 (1H, d, *J* = 5.1Hz, H-6), 5.16 (1H, dd, *J* = 15.2, 8.7 Hz, H-22), 5.02 (1H, dd, *J* = 15.2, 8.7 Hz, H-23), 3.85 (1H, s, H-7), 3.59 (1H, m, H-3), 1.03 (3H, d, *J* = 6.6 Hz, H-21), 1.00 (3H, s, H-19), 0.70 (3H, s, H-18). ^13^C-NMR (200 MHz, CDCl_3_) *δ*_C_: 37.0 (C-1), 31.4 (C-2), 71.4 (C-3), 42.0 (C-4), 146.2 (C-5), 123.9 (C-6), 65.3 (C-7), 37.5 (C-8), 42.3 (C-9), 37.5 (C-10), 20.7 (C-11), 39.1 (C-12), 42.3 (C-13), 49.4 (C-14), 24.4 (C-15), 28.9 (C-16), 55.7 (C-17), 11.8 (C-18), 18.2 (C-19), 40.5 (C-20), 21.2 (C-21), 138.2 (C-22), 129.3 (C-23), 51.2 (C-24), 31.9 (C-25), 19.0 (C-26), 21.1 (C-27), 25.4 (C-28), 12.3 (C-29).

**Compound 20.** C_28_H_44_O_3_, ESI-MS: *m*/*z* 451 [M + Na]^+^. ^1^H-NMR (500 MHz, CDCl_3_) *δ*_H_: 6.50 (1H, d, *J* = 8.5 Hz, H-7), 6.24 (1H, d, *J* = 8.5 Hz, H-6), 3.96 (1H, s, H-3), 0.99 (3H, d, *J* = 6.5, H-21), 0.90 (3H, d, *J* = 6.5 Hz, H-28), 0.88 (3H, s, H-19), 0.82 (3H, d, *J* = 8.5 Hz, H-26), 0.81 (3H, d, *J* = 8.5 Hz, H-27), 0.80 (3H, s, H-18). ^13^C-NMR (125 MHz, CDCl_3_) *δ*_C_: 34.7 (C-1), 30.1(C-2), 66.5 (C-3), 36.9 (C-4), 82.2 (C-5), 135.4 (C-6), 130.7 (C-7), 79.4 (C-8),51.1 (C-9), 36.9 (C-10), 23.4 (C-11), 39.3 (C-12), 44.6 (C-13), 51.7 (C-14), 20.6 (C-15), 28.7 (C-16), 56.2 (C-17), 12.9 (C-18), 18.2 (C-19), 39.7 (C-20), 20.9 (C-21), 135.2 (C-22), 132.3 (C-23), 42.8 (C-24), 33.1 (C-25), 19.6 (C-26), 20.0 (C-27), 17.6 (C-28).

### 2.2. Antioxidant Activities of Methanolic Extract, Fractions and Isolated Compounds

The methods commonly used to determine the antioxidant capacity of substances include DPPH^+^, ABTS^+^, FRAP, and TRAP methods [43], but none of them can comprehensively evaluate the total antioxidant capacity of tested substances. Therefore, the crude methanol extract (CME), different fractions, and isolated compounds were evaluated for their antioxidant activities according to DPPH^+^, ABTS^+^, and FRAP assays. As shown in Table 1, the antioxidant activities of CME and different fractions showed consistent results in three different patterns. The CME had antioxidant activity (IC_50_ = 203.80 ± 21.70 μg/mL for DPPH, 0.61 ± 0.0067 mmol Trolox/g for ABTS, 0.29 ± 0.0051 mmol Trolox/g for FRAP). This result supported the benefits of using *C. citratus* in food and tea. The n-butanol (n-BuOH) fraction (IC_50_ = 41.60 ± 3.09 μg/mL for DPPH, 1.20 ± 0.013 mmol Trolox/g for ABTS, 0.82 ± 0.016 mmol Trolox/g for FRAP) showed the most potent antioxidant activity (*p* < 0.05), and the ethyl acetate (EtOAc) fraction (IC_50_ = 130.70 ± 8.45 μg/mL for DPPH, 0.96 ± 0.0050 mmol Trolox/g for ABTS, 0.42 ± 0.021 mmol Trolox/g for FRAP) showed moderate antioxidant activity (*p* < 0.05). In contrast, the petroleum ether (PE) fraction and aqueous fraction (AF) had no antioxidant activities. The potent antioxidant properties of n-BuOH and EtOAc fractions might be due to the antioxidant and free radical scavenging activities of their flavonoid and phenolic compounds [44]. The results suggested that the main antioxidant compounds of *C. citratus* might present in n-BuOH and EtOAc fractions.

Polyphenols and flavonoids are considered to have potential antioxidant activities [45]. Thus, the isolated compounds **1**–**10** and **12** were screened for their antioxidant activities. As shown in Table 1, compounds **5**, **8**, **9**, and **12** exhibited stronger antioxidant activities (IC_50_ = 7.28–14.81 μM for DPPH, 1.70–2.15 mol Trolox/mol for ABTS, 1.31–2.42 mol Trolox/mol for FRAP) than ascorbic acid. These results indicated that compounds **5**, **8**, **9**, and **12** had a potent free radical scavenging ability and ferric reducing power, which might be developed into effective natural antioxidants. Compounds **1**–**4**, **6**–**7**, and **10** had no significant activity when IC_50_ values were greater than 80 μM for DPPH and were not detected to be active for FRAP at 20 μM. Compared with compound **7**, compounds **8** and **9** exhibited higher DPPH^+^ scavenging activities and ferric reducing power, which revealed that the phenolic hydroxyl groups at C-3, 3″ might contribute to the DPPH^+^ scavenging and ferric reducing abilities. Compound **5** showed more potent antioxidant activities than compound **4**, which suggested that the hydroxyl group at C-3 could enhance ferric reducing and DPPH^+^ scavenging abilities. Interestingly, compounds **2**–**10** and **12** all showed significant ABTS^+^ scavenging abilities, which reminds us that we should pay more attention to the antioxidant activities of polyphenols and flavonoids in *C. citratus*.

### 2.3. α-Glucosidase Inhibition Activity

**Figure 2:**
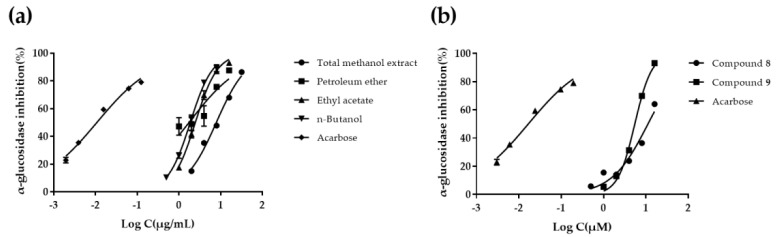
The *α*-glucosidase inhibitory effects of *C. citratus* extracts, and compounds **8**, **9**. (**a**) Concentration−response relationship for *C. citratus* extracts. (**b**) Concentration−response relationship for compounds **8** and **9**. Log C is the logarithm of concentration in μg/mL for *C. citratus* extracts, and in μM for acarbose and compounds **8**, **9** (three independent assays performed in duplicate).

*α*-Glucosidase inhibitors can delay carbohydrate absorption and reduce postprandial hyperglycemia by inhibiting *α*-glucosidase in upper intestinal epithelial cells and reducing glucose conversion. The results of CME and different fractions are shown in Table 2 and Figure 2a. The PE and EtOAc fractions showed the most potent *α*-glucosidase inhibition activities (*p* < 0.05) with IC_50_ value of 1.77 ± 0.55 μg/mL and 2.47 ± 0.10 μg/mL, respectively. The results are consistent with the previous report [13]. The CME and n-BuOH fractions showed moderate *α*-glucosidase inhibition activities (*p* < 0.05) with IC_50_ value of 7.90 ± 0.55 μg/mL and 7.49 ± 0.34 μg/mL, respectively. The AF exhibited no significant *α*-glucosidase inhibitory activity with IC_50_ value > 300 μg/mL. The results indicated that most *α*-glucosidase inhibitory substances should be small polar compounds present in PE and EtOAc fractions. At the same time, our study further provides scientific evidence for the efficacy of *C. citratus* as a herbal medicine in the treatment of diabetes, and demonstrates that the possible pathway is through inhibiting *α*-glucosidase activity.

It has been reported in the literature that flavonoid and polyphenols compounds have hypoglycemic activity [46,47]. Therefore, compounds **1**–**10** and **12** were screened for their *α*-glucosidase inhibitory activities. The results are shown in Table 3. The inhibitory rates of compounds **8** and **9** on *α*-glucosidase were 66.96% and 88.85%, respectively, while compounds **1**–**7, 10** and **12** were less than 50% at 20 μM. Further tests of *α*-glucosidase inhibitory activities on compounds **8** and **9** were carried out, and the results are shown in Table 2 and Figure 2b. Compounds **8** and **9** showed obvious *α*-glucosidase inhibitory activities with IC_50_ values of 11.45 ± 1.82 μM and 5.46 ± 0.25 μM, respectively. This is the first report of the α-glucosidase inhibitory activities of compounds **8** and **9**. The α-glucosidase inhibitory activities of compounds **7**, **8**, and **9** suggested that the phenolic hydroxyl groups at C-3, 3″ might determine whether these types of compounds have α-glucosidase inhibitory activity.

### 2.4. Molecular Docking Study

**Figure 3:**
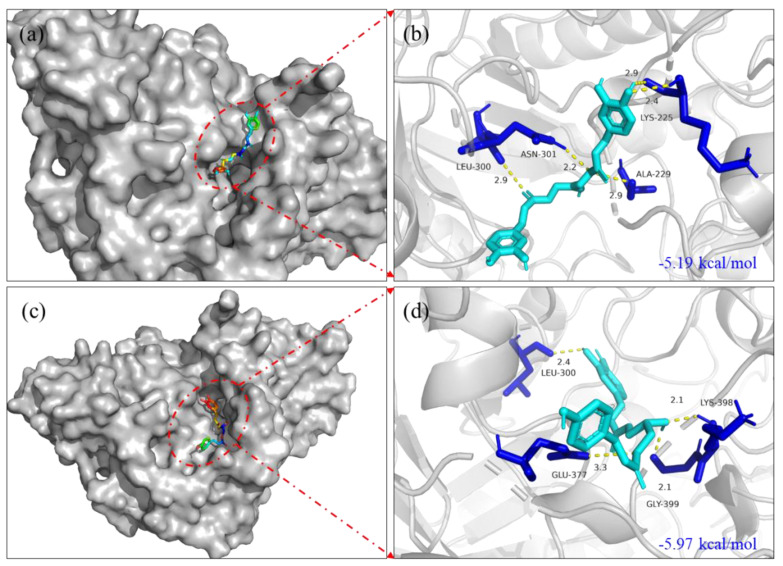
Molecular docking pictures of 1-*O*-p-coumaroyl-3-*O*-caffeoylglycerol (**8**) and 1,3-*O*-dicaffeoylglycerol (**9**) on *α*-glucosidase of 3WY1. (**a**) The surface structure of 3WY1−1,3-*O*-dicaffeoylglycerol. (**b**) The binding site structure of 3WY1−1,3-*O*-dicaffeoylglycerol. (**c**) The surface structure of 3WY1−1-*O*-p-coumaroyl-3-*O*-caffeoylglycerol. (**d**) The binding site structure of 3WY1−1-*O*-p-coumaroyl-3-*O*-caffeoylglycerol.

The molecular docking results showed that 1-*O*-p-coumaroyl-3-*O*-caffeoylglycerol (**8**) and 1,3-*O*-dicaffeoylglycerol (**9**) possessed superior binding energy with *α*-glucosidase (binding energy: −5.19, −5.97 kcal/mol, respectively) (Figure 3.). The surface structures of the ligand−enzyme complex showed that the candidates positioned in the active pocket of *α*-glucosidase, as illustrated in Figure 3a,c. The results indicated that compound **8** formed two hydrogen-bonding interactions with GLY399, LYS398 residues of the enzyme; the distances were 2.1 and 2.1 Å, respectively. Compound **9** formed a hydrogen-bonding interaction with ASN301 residue of *α*-glucosidase, and the distance was 2.2 Å. In summary, the results indicated that candidates might bind to the active site of *α*-glucosidase to inhibit the activity of the enzyme.

### 2.5. Glucose Uptake and Cell Viability

**Figure 4:**
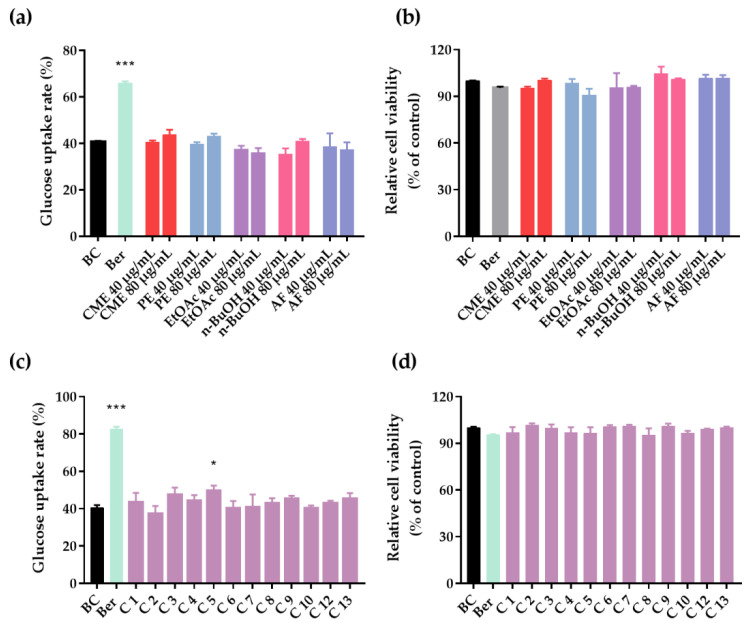
Glucose uptake and cell viability in 3T3-L1 adipocytes. (**a**) Glucose uptake rates of *C. citratus* extract and fractions (CME, PE, EtOAc, n-BuOH, and AF). (**b**) Relative cell viability of *C. citratus* extract and fractions. (**c**) Glucose uptake rates of compounds **1**–**10** and **12**–**13**. (**d**) Relative cell viability of compounds **1**–**10** and **12**–**13**. BC, blank control; Ber, berberine (positive control); C, compound; Compounds at 20 μM and berberine at 10 μg/mL; Data are showed as mean ± SD of three independent experiments; * *p* < 0.05, *** *p* < 0.001 versus blank control.

To examine the effects of the CME, different fractions and isolated compounds of *C. citratus* involved with glucose uptake in 3T3-L1 adipocytes were evaluated. As shown in Figure 4a, the insulin group noticeably promoted the glucose uptake rates of 3T3-L1 adipocytes compared to the control group with a statistically significant difference (*p* < 0.001). It is a pity that compared with the control group, the CME, PE fraction, EtOAc fraction, n-BuOH fraction and AF did not significantly promote glucose uptake in 3T3-L1 adipocytes at 40 and 80 μg/mL (*p* > 0.05). The results showed that the main hypoglycemic effect of *C. citratus* may not be achieved by improving glucose uptake in 3T3-L1 adipocytes. As shown in Figure 4c, compound **5** had a weak effect on glucose uptake in 3T3-L1 adipocytes at 20 μM (*p* < 0.05). Additionally, the results of cell viability (Figure 4b,d) showed that compared with the blank control group, the CME, PE fraction, EtOAc fraction, n-BuOH fraction, AF fraction, and isolated compounds had no cytotoxicity.

## 3. Materials and Methods

### 3.1. Plant Material

The dried aerial part of *Cymbopogon citratus* (D.C.) Stapf was collected in Jinghong in 2018 and identified by Prof. Yumei Zhang. The voucher specimen (No. 2018006) was deposited in the Innovative Drug Research Group, Xishuangbanna Tropical Botanical Garden, Chinese Academy of Sciences.

### 3.2. Chemicals, Reagents and Cell

NMR was determined by the BrukerAM-400 MHz and Bruker DRX-500 MHZ AVANCE III-600 MHz (Bruker Corporation, Madison, WI, USA) (TMS as internal standard) NMR instrument. EI-MS was determined by the VG AutoSpec 3000 mass spectrometer. Semi-preparative HPLC was run on a Waters e2695 system (Waters Corporation, Milford, MA, USA) with a Waters 2487 detector and an Agilent Zorbax SB-C18 column (250 mm × 9.4 mm, 5 μm) (Agilent Corporation, Santa Clara, CA, USA). Column chromatography was conducted with silica gel (Qingdao Marine Chemical Co. Ltd., Qingdao, China), RP-C18 (Merck KGaA, Darmstadt, Germany), and Sephadex LH-20 (Cytiva Sweden AB, Upsala, Sweden). DPPH (2,2-diphenyl-1-picrylhydrazyl) was purchased from Macklin Biochemical Co. Ltd. (Shanghai, China). FRAP and ABTS^+^ assays were evaluated by a commercial assay kit (Suzhou Comin Biotechnology Co., Ltd., Suzhou, China). *α*-Glucosidase (25.4 U/mg), acarbose, 4-nitrophenyl-*α*-D-glucopyranoside (pNPG), and ascorbic acid were purchased from Yuanye Biotechnology Co., Ltd. (Shanghai, China). The other chemicals and reagents were purchased from local suppliers. The absorbance was measured by a microplate reader (Molecular Devices, Palo Alto, Santa Clara, CA, USA). The 3T3-L1 mouse preadipocytes were purchased from the American Type Culture Collection (ATCC, Manassas, VA, USA). High glucose DMEM, low glucose DMEM, Pen-Strep solution (P/S), insulin, certified fetal bovine serum (FBS), special newborn calf serum (NBCS), and phosphate buffered saline (PBS) were purchased from Biological Industries (Shanghai, China). 3-isobutyl-1-methylxanthine (IBMX), and dexamethasone (DEX) were obtained from Sigma-Aldrich (St. Louis, MO, USA). The glucose test kit was purchased from Rongsheng Biotech Co., Ltd. (Shanghai, China). Rosiglitazone (ROSI) was purchased from Meilun Biotech Co., Ltd. (Dalian, Liaoning, China). Dimethyl sulfoxide (DMSO) was obtained from Solarbio (Beijing; China). CellTiter 96^®^ AQueous One Solution Cell Proliferation Assay was also acquired (Promega Corporation, Madison, WI, USA).

### 3.3. Extraction, Isolation, and Purification

The dried aerial part (18 kg) of *C. citratus* was powdered and then extracted with industrial methanol three times at room temperature (7 days, 3 days, and 3 days, respectively). The CME (3.02 kg) was obtained by concentration under vacuum, subsequently dissolved by stirring in hot water, and successively partitioned with PE, EtOAc, and n-BuOH, to afford the PE fraction (463.6 g), EtOAc fraction (180.0 g), and n-BuOH fraction (226.1 g).

The PE fraction was subjected to a silica gel column (200–300 mesh, 4 kg) using the gradient elution manner with PE/acetone (10:1, 7:1, 5:1, 2:1, 1:1, 0:1) to gain 10 fractions (Fr.1- Fr.10). Fraction 2 was separated using Sephadex LH-20 column chromatography and recrystallized to gain **17** (20 mg). Fraction 3 was subjected to a silica gel column and further purified by Sephadex LH-20 column chromatography to obtain **20** (7 mg). Fraction 5 was subjected to silica gel column and further purified using Sephadex LH-20 column chromatography to obtain **10** (17 mg), **13** (9 mg), and **14** (37 mg). Fraction 6 was subjected to a silica gel column and further purified using Sephadex LH-20 column chromatography to obtain **16** (9 mg) and **19** (7 mg).

The EtOAc fraction was separated on a silica gel column (200–300 mesh, 1.5 kg) using the gradient elution manner with CHCl_3_/MeOH (30:1, 15:1, 9:1, 4:1, 2:1, 0:1) to obtain 12 fractions (Fr.11- Fr.22). Fraction 15 was subjected to a silica gel column and further purified using Sephadex LH-20 column chromatography and recrystallizing to obtain **1** (8 mg), **4** (13 mg). Fraction 18 was subjected to a silica gel column and further purified using Sephadex LH-20 column chromatography and semi-preparative HPLC to obtain **2** (14 mg). Fraction 19 was subjected to a silica gel column and further purified using Sephadex LH-20 column chromatography to obtain **6** (5 mg). Fraction 20 was subjected to a silica gel column and further purified using Sephadex LH-20 column chromatography to obtain **8** (8 mg). Fraction 22 was subjected to a silica gel column and further purified using Sephadex LH-20 column chromatography and semi-preparative HPLC to obtain **7** (9 mg). Fraction 22 was subjected to a silica gel column and further purified using Sephadex LH-20 column chromatography and semi-preparative HPLC to obtain **5** (6 mg), **9** (7 mg), **11** (9 mg).

The n-BuOH fraction was separated on silica gel column (200–300 mesh, 1.5 kg) using the gradient elution manner with CHCl_3_/MeOH (9:1, 5:1, 3:1, 0:1) to obtain 9 fractions (Fr.23- Fr.31). Fraction 24 was subjected to a silica gel column and further purified by Sephadex LH-20 column chromatography and recrystallizing to obtain **12** (5 mg). Fraction 25 was subjected to a silica gel column and further purified using Sephadex LH-20 column chromatography and recrystallizing to obtain **3** (6 mg). Fraction 27 was subjected to a silica gel column and further purified using Sephadex LH-20 column chromatography to obtain **15** (11 mg). Fraction 30 was subjected to a silica gel column and further purified using Sephadex LH-20 column chromatography to obtain **18** (24 mg).

### 3.4. Measurement of Antioxidant Activity

The antioxidant activities of the crude extracts and isolated compounds were evaluated by implementing 2,2-diphenyl-1-picrylhydrazyl free radical scavenging (DPPH^+^), 2,2’-azino-bis (3-ethylbenzothiazoline-6-sulphonic free radical scavenging (ABTS^+^), and the ferric reducing antioxidant power (FRAP) assay.

The DPPH^+^ assay was measured according to the previous report [48] with slight modifications. Briefly, the crude extracts and chemical compounds were dissolved in anhydrous ethanol at concentrations ranging from 100 to 3200 μg/mL and 20 to 800 μM. The DPPH^+^ was dissolved with anhydrous methanol to 0.1 mM.

Then 180 μL DPPH solution and 20 μL sample were mixed in each well of a 96-well plate and incubated for 30 min at room temperature in the dark. The absorbance was recorded at 517 nm using a microplate reader. Ascorbic acid was used as a positive control. The DPPH^+^ scavenging activity was calculated according to the following formula:

Inhibition (%) = [1 − (A_1_ − A_2_)/(A_3_ − A_4_)] × 100 (1)

A_1_ = absorbance of the sample group with DPPH, A_2_ = absorbance of the sample control group without DPPH, A_3_ = absorbance of the control group with DPPH, and A_4_ = the absorbance of the blank control.

ABTS^+^ and FRAP assays were measured by commercial assay kits (Suzhou Comin Biotechnology Co., Ltd., Suzhou, China). The experimental method was determined according to the method used in the literature [48]. The crude extracts and chemical compounds were dissolved in anhydrous ethanol at concentrations of 800 μg/mL and 40 μM. The absorbance was measured using a microplate reader at 734 nm in the dark and 593 nm after reaction for 20 min in the dark, respectively. Trolox was used as a standard reference compound to quantitate ABTS^+^ and FRAP capacity. The results were expressed in µmol Trolox/g or mol Trolox/mol. The standard curve was drawn using Trolox solutions at a range of concentrations.

### 3.5. α-Glucosidase Inhibition Assay

The *α*-glucosidase inhibitory activity was measured according to the procedure described in the paper [49], with slight modifications. Briefly, add 50 μL *α*-glucosidase (0.1 U/mL) and 80 μL phosphate buffered saline (PBS) (pH = 6.9) to each well in 96-well plates, then add 10 μL sample solution (crude extracts and chemical compounds at concentrations ranging from 200 to 6400 μg/mL and 10 to 400 μM) and incubate at 37 °C for 15 min. Immediately, 40 μL PNPG (2.5 mM) was added to each well and reacted at 37 °C for 30 min. In the end, 20 μL Na_2_CO_3_ (0.5 mol/L) was added to stop the reaction, and then the absorbance value was measured using a microplate reader at 405 nm. The total volume of the reaction system was 200 μL. The *α*-glucosidase inhibition activity was calculated according to the following formula:

Inhibition (%) = [1 − (A_1_ − A_2_)/(A_3_ − A_4_)] × 100 (2)

A_1_ = the absorbance of the sample group with *α*-glucosidase, A_2_ = the absorbance of the sample control group without *α*-glucosidase, A_3_ = the absorbance of the control group without samples, and A_4_ = the absorbance of the blank control without samples and *α*-glucosidase.

Results were expressed as IC_50_ values calculated according to Prism7.0 software.

### 3.6. Molecular Docking

Molecular docking was applied to identify the possible binding sites between candidates and *α*-glucosidase according to a previous study [50]. The crystal structure of halomonas *α*-glucosidase (PDB ID: 3WY1) was obtained from the Protein Data Bank (PBD), and the three-dimensional structures of the candidates were established through MarvinSketch. The water molecules of *α*-glucosidase were removed via the PyMOL molecular graphics system (version: 2.2.0) to obtain a stable *α*-glucosidase structure. AutoDock Tools (ADT, version: 1.5.6) was used to accomplish molecular docking in silico [51]. The cubic grid box dimensions of *α*-glucosidase were defined as x = 96, y = 98, and z = 118 Å with spacing of 0.686 Å. Finally, the PyMOL molecular graphics system (version: 2.2.0) was used to visualize ligand-enzyme interactions. Based on the minimum energy scoring, the best binding conformations between *α*-glucosidase and the candidates were selected from all docking results [52].

### 3.7. 3T3-L1 Preadipocytes Culture and Differentiation

The 3T3-L1 preadipocytes were cultured and differentiated using the method described earlier with slight modifications [53]. The 3T3-L1 preadipocytes were cultured in high glucose DMEM supplemented with 10% NBCS, 1% P/S, and then starved until the cells reached confluence (day 0). Two days later (day 2), the cells were induced for differentiation with high glucose DMEM supplemented with 10% FBS, 1% P/S, IBMX, DEX, Rosi, and insulin. On day 5, the medium was changed to high glucose DMEM containing 10% FBS, 1% P/S, and 100 nM insulin for one day (day 6). Then the cells were completely differentiated into mature adipocytes.

### 3.8. Glucose Uptake and Cell Viability Assay

The glucose uptake test was carried out according to the procedure described in the paper [53]. The 3T3-L1 adipocytes were inoculated in a 96-well plate at 5 × 10^4^ cells/well for 24 h. Then the berberine (10 μg/mL) and samples (40 μg/mL, 80 μg/mL or 20 μM) were added to individual 3T3-L1 adipocytes and repeated 3 times. After 48 h of culture, the glucose uptake was measured according to the operating instructions of the glucose content determination kit. Following the glucose uptake test, the cell viability was determined using the CellTiter 96^®^ aqueous cell proliferation test [54]. Then 15 μL of CellTiter 96^®^ AQueous One Solution Cell Proliferation Assay reagent was added to each well. Then the absorbance was measured using microplate reader at 490 nm after incubation at 37 °C for 4 h. The cell viability was calculated according to the following formula:

Cell viability rate (%) = A_1/_A_2_ × 100 (3)

A_1_ = the absorbance of the blank control, A_2_ = the absorbance of each fraction group, each compound group, or positive control group.

### 3.9. Statistical Analysis

Each experiment’s measurements were repeated there times, and all the data were expressed as mean ± standard deviation (SD). The IC_50_ of DPPH^+^ scavenging activity and *α*-glucosidase inhibition activity were calculated according to non-linear regression analysis. The differences between different samples were assessed by using a one-way analysis of variance (ANOVA). If the *P* value was less than 0.05, the data of samples were considered statistically significant. All analyses were carried out using Graphpad Prism7.0 software (GraphPad Software Inc., San Diego, CA, USA).

## 4. Conclusions

In this study, 20 compounds, including terpenoids, glycerides, flavones, and their glycosides, aromatic ketones, phenolic acids, alkaloids, and steroids, were isolated from the dried aerial part of *C. citratus*. Among them, compounds **1**–**3**, **6**–**11**, **13**–**16**, and **18**–**20** were reported for the first time from this species. The bioactive investigations of the ethanol extract and different fractions of *C. citratus* exhibited that the EtOAc and n-BuOH fractions had a potent antioxidant effect, and the EtOAc and PE fractions had a potent *α*-glucosidase inhibitory effect. However, we did not find compounds with *α*-glucosidase inhibitory activity in the PE fraction, so it would be valuable to study the bioactive compounds in the PE fraction extensively. Compounds **8** and **9** showed a potent antioxidant effect and an *α*-glucosidase inhibitory effect. The *α*-glucosidase inhibitory activities of compounds **8** and **9** were reported for the first time. Furthermore, the results revealed that compounds **8** and **9** might be developed as candidate drugs to cure diabetes because of their potential regulatory roles on oxidative stress and digestive enzyme. This study enriched the chemical composition diversity of *C. citratus* and provided effective evidence for its use in health food and hypoglycemic herbal medicine.

## Figures and Tables

**Table 1:** Antioxidant activities of extracts and compounds **1**–**10, 12** from *C. citratus*.

Samples	DPPH Assay	ABTS Assay ^B^	FRAP Assay ^C^
Extracts	IC_50_ (μg/mL)	mmol Trolox/g	mmol Trolox/g
CME	203.80 ± 21.70 ^a^	0.61 ± 0.0067 ^c^	0.29 ± 0.0051 ^c^
PE	>320.00	0.12 ± 0.0082 ^d^	0.042 ± 0.0045 ^d^
EtOAc	130.70 ± 8.45 ^b^	0.96 ± 0.0050 ^b^	0.42 ± 0.021 ^b^
n-BuOH	41.60 ± 3.09 ^c^	1.20 ± 0.013 ^a^	0.82 ± 0.016 ^a^
AF	>320.00	0.13 ± 0.034 ^d^	0.076 ± 0.011 ^d^
Compounds	IC_50_ (μM)	mol Trolox/mol	mol Trolox/mol
**1**	>80.00	2.40 ± 0.16 ^a^	n.d.
**2**	>80.00	0.87 ± 0.11 ^f^	n.d.
**3**	>80.00	1.97 ± 0.079 ^cd^	n.d.
**4**	>80.00	2.28± 0.10 ^ab^	n.d.
**5**	7.41 ± 0.74 ^c^	2.15 ± 0.0619 ^abc^	1.73 ± 0.080 ^b^
**6**	>80.00	1.80 ± 0.0462 ^de^	0.012 ± 0.014 ^d^
**7**	>80.00	1.99 ± 0.11 ^cd^	n.d.
**8**	14.81 ± 1.83 ^b^	1.70 ± 0.066 ^e^	1.42 ± 0.073 ^c^
**9**	8.82± 1.12 ^c^	2.065 ± 0.050 ^bcd^	2.42 ± 0.10 ^a^
**10**	>80.00	1.64 ± 0.14 ^e^	n.d.
**12**	7.28 ± 1.48 ^c^	1.86 ± 0.075 ^de^	1.31 ± 0.057 ^c^
Ascorbic acid ^A^	19.81 ± 1.27 ^a^	1.66 ± 0.050 ^e^	1.68 ± 0.063 ^b^

Data were expressed as the mean value ± SD (*n* = 3); Means followed by the different superscript letters (a–f) are significantly different (*p* < 0.05); IC_50_: half inhibition concentration; ^A^ Positive control (DPPH^+^ assay, ABTS^+^, and FRAP assay); ^B^ The ABTS and ^C^ the FRAP values mean that each gram of sample corresponds to the number of millimoles of Trolox or each mole of sample corresponds to the number of moles of Trolox at the same absorbance.

**Table 2:** *α*-Glucosidase inhibitory activities of extracts and compounds **8**–**9** from *C. citratus*.

Samples	*α*-Glucosidase Inhibitory Activity
Extracts	IC_50_ (μg/mL)
CME	7.90 ± 0.55 ^a^
PE	1.77 ± 0.55 ^b^
EtOAc	2.47 ± 0.10 ^b^
n-BuOH	7.49 ± 0.34 ^a^
AF	>320.00
Compounds	IC_50_ (μM)
**8**	11.45 ± 1.82 ^a^
**9**	5.46 ± 0.25 ^b^
Acarbose ^A^	0.017 ± 0.0020 ^c^

Data were expressed as the mean value ± SD (*n* = 3); Means followed by the different letters (a–c) are significantly different (*p* < 0.05); IC_50_: half inhibition concentration; ^A^ Positive control (*α*-Glucosidase inhibitory effect); n.d., not determined.

**Table 3:** *α*-Glucosidase inhibitory activities of compounds **1**–**10** and **12**.

Compounds	Inhibitory Rate (%) ^A^
**1**	n.d.
**2**	22.6 ± 3.4
**3**	n.d.
**4**	n.d.
**5**	n.d.
**6**	5.3 ± 1.8
**7**	46.1 ± 2.4
**8**	67.0 ± 4.2
**9**	88.8 ± 0.4
**10**	20.8 ± 7.3
**12**	21.9 ± 6.0
Acarbose	99.4 ± 0.0

Data were expressed as the mean value ± SD (*n* = 3); ^A^ Percent inhibition at a concentration of 20 μM; n.d., not determined.

## Data Availability

All data presented in this study are available in the article.

## Sample Availability

Samples of the compounds are not available from authors.
